# Black tea consumption and cancer risk: a prospective study.

**DOI:** 10.1038/bjc.1986.226

**Published:** 1986-10

**Authors:** L. K. Heilbrun, A. Nomura, G. N. Stemmermann

## Abstract

In a prospective cohort study, men of Japanese ancestry were clinically examined from 1965 to 1968. For 7,833 of these men, data on black tea consumption habits were recorded. Since 1965, newly diagnosed cancer incidence cases have been identified: 152 colon, 151 lung, 149 prostate, 136 stomach, 76 rectum, 57 bladder, 30 pancreas, 25 liver, 12 kidney and 163 at other (miscellaneous) sites. Compared to almost-never drinkers, men habitually drinking black tea more than once/day had an increased relative risk (RR) for rectal cancer (RR = 4.2). This positive association (P = 0.0007) could not be accounted for by age or alcohol intake. We also observed a weaker but significant negative association of black tea intake and prostate cancer incidence (P = 0.020). There were no significant associations between black tea consumption and cancer at any other site.


*Br. J. Cancer* (1986), **54**, 677–683

### Black tea consumption and cancer risk: A prospective study

L.K. Heilbrun, A. Nomura & G.N. Stemmermann

*Japan–Hawaii Cancer Study, Kuakini Medical Center, 347 N. Kuakini Street, Honolulu, HI 96817, USA.*

**Summary** In a prospective cohort study, men of Japanese ancestry were clinically examined from 1965 to 1968. For 7,833 of these men, data on black tea consumption habits were recorded. Since 1965, newly diagnosed cancer incidence cases have been identified: 152 colon, 151 lung, 149 prostate, 136 stomach, 76 rectum, 57 bladder, 30 pancreas, 25 liver, 12 kidney and 163 at other (miscellaneous) sites. Compared to almost-never drinkers, men habitually drinking black tea more than once/day had an increased relative risk (RR) for rectal cancer (RR = 4.2). This positive association ($P = 0.0007$) could not be accounted for by age or alcohol intake. We also observed a weaker but significant negative association of black tea intake and prostate cancer incidence ($P = 0.020$). There were no significant associations between black tea consumption and cancer at any other site.

Tea is the second most commonly consumed beverage in the world (Wickremasinghe, 1978), next to water. Tea originated in China as far back as 2737 B.C., although its earliest written mention was in 350 B.C. in a Chinese dictionary (Wickremasinghe, 1978). Tea was brought to Europe in 1559 A.D. The major types of tea are distinguished by their processing methods. Black tea is made from leaves that have been withered before being rolled and dried (Bokuchava & Skobeleva, 1980). Quantitatively, black tea is the major type of tea produced worldwide (Wickremasinghe, 1978).

Of Western nations, the UK has the highest annual per capita tea consumption of any country: 4.38 kg per year in 1965–1966 (Stocks, 1970), and 3.86 kg per year in 1975 (Bokuchava & Skobeleva, 1980). In contrast, the US per capita tea consumption levels are 0.33 kg per year in each time period. After the UK, per capita tea consumption is also very high in several English-speaking countries: Ireland (4.25 kg per year), New Zealand (3.35), Australia (2.30), and Canada (1.11), based on the 1965–1966 data. In spite of its frequent consumption, relatively little data has been published on the relationship of tea intake to the risk of cancer.

Brewed black tea has caused skin cancers in mice when applied to the neck (Kaiser, 1967). Tannic acid, a substance in tea leaves, has been shown to produce tumours of the liver and bile ducts in rats (Korpassi & Mosonyi, 1950). Condensed tannins have produced sarcomas at the injection site and liver tumours in rats and mice, while extracts of hydrolysable tannins have caused liver tumours in mice (Kirby, 1960). The tannin-containing fraction of black tea has produced histiocytomas at the injection site in rats (Kapadia *et al.*, 1976). These animal studies have generally used high doses. A low dose study of tannic acid showed no liver damage or liver cancer in mice, and only a slight excess of other cancers compared to the control groups (Bichel & Bach, 1968). The animal data are therefore somewhat inconsistent, and each study typically spanned only 3–10 months of exposure.

Black tea was found to be mutagenic according to the Ames test (Nagao *et al.*, 1979). They also noted that the mutagenicity from one cup of tea was more than that from the smoke condensate of one cigarette.

In epidemiologic studies, tea drinking was positively associated with the risk of renal pelvis cancer (McLaughlin *et al.*, 1983) and kidney cancer (McLaughlin *et al.*, 1984) for women, but not men. A recent report shows increased pancreatic cancer risk among heavier tea consumers for both men and women (Kinlen & McPherson, 1984). A very large case-control study showed a slight increase in bladder cancer risk among heavier female tea drinkers ($\geqq 1$ cup/day) but not so for the males (Hartge *et al.*, 1983). Other authors have not found an association between tea intake and the risk of bladder cancer (Morgan & Jain, 1974; Miller *et al.*, 1978; Howe *et al.*, 1980; Sullivan, 1982), pancreatic cancer (MacMahon *et al.*, 1981), or kidney cancer (Armstrong *et al.*, 1976). Rectal cancer was not significantly associated with (black) tea intake in two studies (Phillips & Snowdon, 1985; Tajima & Tominaga, 1985), although colon cancer has shown a positive association (Tajima & Tominaga, 1985; Stocks, 1970).

In view of these discrepant findings, and the sometimes crude classification of tea consumption (ever *vs.* never drank), we have utilized our existing prospective study to examine the relationship of black tea intake and cancer risk. More than 7,800

Correspondence: L.K. Heilbrun.
Received 2 January 1986; and in revised form 26 June 1986.

© The Macmillan Press Ltd., 198

men have been followed for at least 16 years for the development of any type of cancer. We are unaware of any other prospective study focused on tea intake and cancer in a cohort of this size.

### Methods

A cohort of 8006 Japanese men, residing on the Hawaiian island of Oahu, and born in the years 1900 to 1919, was clinically examined from 1965 to 1968 (Worth & Kagan, 1970). During the interview, the men were asked about their habitual frequency of consumption of black tea (Tillotson *et al.*, 1973). This was recorded in five categories: almost never, <twice per week, 2–4 times per week, almost daily (5–7 times per week), >once per day.

Since the examination of these men, newly diagnosed cases of cancer have been identified by continuous surveillance of the major hospitals on Oahu, augmented by linkage with the Hawaii Tumor Registry. We estimate that only 1.8% of these men have moved from Oahu, so our case-finding is virtually complete. We excluded 81 prevalence cancer cases and 90 suspected incidence cases who were without tissue confirmation. For two men (one lung cancer case, one control), black tea consumption frequency was unknown. This left 7,833 study eligible men for analysis, including 951 tissue confirmed cancer incidence cases as of April, 1985.

During the surveillance period, 17.0% (1328 of 7,833) of the men eligible for analysis had died. Cancer accounted for 436 (32.8%) of the total deaths. Coronary heart disease and stroke deaths numbered 306 (23.0%) and 138 (10.4%), respectively. Due to the relatively low proportion of deaths in our study population, competing risks of death appeared unlikely as a major confounder in our data. The time at risk for each subject was calculated as the number of months from examination to cancer diagnosis, date of death, or April 30, 1985, whichever occurred first. The 7,833 men available for analysis provided 126,613 person-years at risk after deaths were accounted for.

Age-adjusted proportions of frequent (almost daily or >once per day) consumers of black tea were computed using a one-way, unbalanced analysis of covariance (Freund & Littell, 1981). The relative risk (RR) of site-specific cancer associated with tea intake was derived from proportional hazards regression models (Cox, 1972), while adjusting for age at examination and other selected covariates. Men in the lowest black tea consumption category (almost never) were chosen as the referent group, and were assigned RR=1.0. Indicator (0, 1) variables were used to denote membership of subjects in their respective tea intake categories. The natural antilogarithm of the Cox model coefficient for a given black tea consumption category estimated the RR of that site-specific cancer relative to men in the referent group. Rectal cancer risk was adjusted for age and habitual alcohol intake (oz. per month of ethanol) since alcohol intake is also a risk factor for rectal cancer in our cohort (Pollack *et al.*, 1984). Lung, bladder and pancreatic cancer risk was adjusted for pack-years of cigarette smoking (whether current or past) as well as age. Covariate-adjusted 95% confidence intervals were used to determine whether an adjusted relative risk was significantly different from unity. Tests for linear trend in the log of the relative hazard (Lee, 1980), were obtained from Cox models using the ungrouped black tea variable (in five levels, coded 0 through 4) and relevant covariates. All Cox models were fitted using iterative maximum likelihood methods (Harrell, 1983).

### Results

Of 7,833 men eligible for analysis, 3,808 (48.6%) were black tea consumers. The age-specific tea intake data (Table I) show that black tea consumption declines with age.

The age-adjusted proportion of frequent black tea consumption by cancer site is shown in Table II. Only rectal cancer cases had a significant excess black tea intake above the level of the controls. Slightly higher tea consumption was also observed for pancreas cancer cases. Lower tea intake was found for prostate, bladder, kidney, and liver cancer cases. Due to the very small number of kidney cancer cases, we did not explore their low tea intake further.

Table III shows the dose-response relationships for these five cancers. Black tea consumption level showed a strong, monotonic relationship to rectal cancer risk. Prostate cancer risk showed a signifi-

**Table I** Black tea intake by age group.

| | | *% Black Tea consumers* | | |
|---|---|---|---|---|
| *Age at exam* | *N* | *at least twice/wk* | *almost daily or >once/day* | *>once/day* |
| 45–49 | 1,819 | 25.8 | 15.8 | 1.7 |
| 50–54 | 2,751 | 25.6 | 16.5 | 2.4 |
| 55–59 | 1,548 | 21.9 | 14.5 | 1.7 |
| 60–64 | 1,295 | 18.8 | 13.6 | 2.3 |
| 65–68 | 420 | 15.0 | 11.7 | 1.9 |
| Total | 7,833 | 23.3 | 15.2 | 2.1 |

**Table II** Age-adjusted proportion of frequent black tea consumption by cancer site.

| *Cancer site* | *N* | *n*[a] | *Age-adjusted proportion* (%)[b] | *P value*[c] |
|---|---|---|---|---|
| Rectum | 76 | 20 | 26.9 | 0.005 |
| Pancreas | 30 | 5 | 17.4 | 0.753 |
| Other sites | 163 | 25 | 15.7 | 0.889 |
| Colon | 152 | 22 | 14.8 | 0.867 |
| Stomach | 136 | 19 | 14.6 | 0.815 |
| Lung | 151 | 21 | 14.3 | 0.736 |
| Prostate | 149 | 15 | 10.8 | 0.134 |
| Bladder | 57 | 6 | 10.7 | 0.342 |
| Kidney | 12 | 1 | 8.4 | 0.508 |
| Liver | 25 | 0 | 0.0 | 0.033 |
| Controls | 6,882 | 1,056 | 15.3 | N/A[d] |

[a]Number of almost daily or more than once per day consumers of black tea; [b]Adjusted for age at exam by analysis of covariance; [c]For comparing each site-specific mean to that of controls; [d]Not applicable.

cant, but somewhat erratic negative association with black tea intake. Pancreatic and bladder cancer associations were erratic and not statistically significant. Liver cancer risk showed a monotonic, negative association with black tea consumption frequency but not significantly so, presumably due to the small number of cases.

The other individual cancer sites had generally unremarkable patterns of risk (not shown), with nonsignificant adjusted RR=0.5–1.5. Those tests for trend were not significant ($P > 0.25$ for each remaining cancer site).

The positive black tea and rectal cancer relationship was confined to the older men (≧58.0 yrs old at examination), who had an adjusted RR=8.7 for the highest intake level *vs*. RR=1.4 in the younger men (see Table IV). Age 58 was used because it separated the rectal cancer cases into two approximately equal groups. In contrast, the negative association of prostate cancer and black tea consumption was not greatly affected by age. In neither age group was the trend in prostate cancer risk statistically significant.

Time from examination to diagnosis had some influence on these two associations as shown in Table V. For rectal cancer cases diagnosed within 10 years, the association with black tea consumption is monotonic, but of lesser magnitude than for cases diagnosed ≧10 years after examination. For the 28 cases diagnosed ≧10 years after examination, the relationship is not monotonic. However, it attains a greater adjusted RR in the highest black tea intake category although this is based on only three cases. The linear trend in rectal cancer risk is significant in either time interval.

The negative prostate cancer association was erratic (and nonsignificant) when only cases diagnosed within 10 years of examination were considered. The 105 cases diagnosed ≧10 years after examination yielded a nearly monotonic negative association with significant trend in risk. This is quite similar to the overall prostate cancer relationship shown in Table III.

### Discussion

It is important to note that most of the published human cancer studies on tea consumption have utilized populations in North America, Great Britain, or other Western countries. In these areas

**Table III** Adjusted relative risks of selected cancers by frequency of consumption of black tea.

| Black tea consumption | No. of controls (*n*=6,882) | Site of cancer: Rectum[a] (*n*=76) | Pancreas[b] (*n*=30) | Prostate[c] (*n*=149) | Bladder[b] (*n*=57) | Liver[a] (*n*=25) |
|---|---|---|---|---|---|---|
| Almost never | 3,479 | 1.0 (31) | 1.0 (18) | 1.0 (95) | 1.0 (28) | 1.0 (15) |
| <twice/wk | 1,773 | 1.3 (17) | 0.6 (4) | 0.8 (34) | 1.4 (18) | 0.8 (6) |
| 2–4 times/wk | 574 | 2.0 (8) | 1.4 (3) | 0.4 (5) | 1.0 (5) | } 0.6 (4) |
| Almost daily | 911 | 2.1[d] (15) | } 0.9 (5) | } 0.6 (15) | } 0.8 (6) | |
| >once/day | 145 | 4.2[d] (5) | | | | |
| *P* value for trend: | | 0.0007 | 0.870 | 0.020 | 0.681 | 0.134 |

Table entries are the adjusted RR, with the number of cases in parentheses. Braces indicate consumption categories combined prior to analysis. [a]RR adjusted for age at examination and alcohol intake (oz. per month); [b]RR adjusted for age at examination and pack-yrs of smoking; [c]RR adjusted for age at examination only; [d]Adjusted RR is significantly different from unity with $P < 0.05$.

**Table IV** Adjusted relative risks of rectal and prostate cancer by age group and by frequency of black tea consumption.

| Black tea consumption | No. of controls age at examination <58 yrs (n=5,017) | No. of controls age at examination ≧58 yrs (n=1,865) | Rectal cancer age at examination <58 yrs (n=37) | Rectal cancer age at examination ≧58 yrs (n=39) | Prostate cancer age at examination <58 yrs (n=66) | Prostate cancer age at examination ≧58 yrs (n=83) |
|---|---|---|---|---|---|---|
| Almost never | 2,341 | 1,138 | 1.0 (15) | 1.0 (16) | 1.0 (39) | 1.0 (56) |
| <twice/wk | 1,385 | 388 | 1.4 (12) | 1.0 (5) | 0.6 (14) | 1.1 (20) |
| 2–4 times/wk | 471 | 103 | 1.4 (4) | 2.9 (4) | 0.5 (4) | 0.2 (1) |
| Almost daily | 710 | 201 | 1.1 (5) | 3.5[a] (10) | } 0.7 (9) | } 0.5 (6) |
| >once/day | 110 | 35 | 1.4 (1) | 8.7[a] (4) | | |
| P value for trend: | | | 0.670 | <0.0001 | 0.160 | 0.062 |

Table entries are the RRs adjusted for the same covariates as in **Table III**, with the number of cases in parentheses. Braces indicate consumption categories combined prior to analysis. [a]Adjusted RR is significantly different from unity with $P<0.05$.

**Table V** Adjusted relative risks of rectal and prostate cancer by time interval from examination to diagnosis and by frequency of black tea consumption.

| Black tea consumption | No. of controls (n=6,882) | Rectal cancer time interval <10 yrs (n=48) | Rectal cancer time interval ≧10 yrs (n=28) | Prostate cancer time interval <10 yrs (n=44) | Prostate cancer time interval ≧10 yrs (n=105) |
|---|---|---|---|---|---|
| Almost never | 3,479 | 1.0 (21) | 1.0 (10) | 1.0 (27) | 1.0 (68) |
| <twice/wk | 1,773 | 1.0 (9) | 1.8 (8) | 1.3 (13) | 0.7 (21) |
| 2–4 times/wk | 574 | 1.5 (4) | 3.0 (4) | Unk.[b](0) | 0.5 (5) |
| Almost daily | 911 | 2.5[a] (12) | 1.3 (3) | } 0.6 (4) | } 0.6 (11) |
| >once/day | 145 | 2.6 (2) | 7.5[a] (3) | | |
| P value for trend: | | 0.008 | 0.034 | 0.232 | 0.043 |

Table entries are the RRs adjusted for the same covariates as in **Table III**, with the number of cases in parentheses. Braces indicate consumption categories combined prior to analysis. [a]Adjusted RR is significantly different from unity with $P<0.05$; [b]RR is not estimable with no cases available.

black tea is probably the most popular type of tea, since 98 percent of the international tea trade is in black tea (New Encyclopaedia Britannica, 1979). Therefore, these past studies were quite likely based on black tea consumption, even though nearly all of them used the non-specific term 'tea'.

The major finding in our study is the positive association of black tea consumption and rectal cancer risk. Very few reports could be found in the literature about this. Phillips and Snowdon (1985) comment that they found no significant relationship between the frequency of tea intake and colorectal cancer mortality risk in a prospective study of 25,493 Seventh Day Adventists. However, no tea data are presented in their report, so it is uncertain how their findings might vary by sex or by site: colon (56 male cases) *vs.* rectum (15 male cases). It is also possible that with only 15 male rectal cancer cases in their study, it was unlikely that they would find an association with tea consumption.

A hospital-based case-control study in Japan (Tajima & Tominaga, 1985) found no significant association between current black tea intake (yes *vs.* no) and rectal cancer (RR=0.93), based on 51 cases. For colon cancer (42 cases), the black tea consumers had a RR=1.70, but that RR was not statistically significant. Exposure contrasts limited to just yes/no categories reduce the chances of finding a meaningful association, especially since no dose-response relationship can be assessed.

A Canadian case-control study (Miller *et al.*, 1983) showed a slightly increased rectal cancer risk (RR=1.2) for men reporting higher intake of 'beverages' (tea, coffee, colas combined). No data were presented or even mentioned for tea alone, so interpretation of this finding is difficult. An international geographic correlation study (Stocks, 1970) showed a very slight negative association with rectal cancer (but a strong positive association with colon cancer). It is well recognized that data from correlational studies are less reliable than data from prospective studies, such as ours. In view of the limitations in study design or in the data presented from other published studies, our findings for rectal cancer still seem tenable. We have shown a strong dose-response relationship, and the positive association persisted over the entire 16–19 years of follow-up which indicates the association may not be spurious.

We could find no published data on tea intake and prostate cancer except for a weak negative association shown in the geographic correlation study (Stocks, 1970). Our data are consistent with that finding and seem more directed at prostate cancer risk 10 or more years after examination.

There was virtually no association of black tea intake and pancreatic cancer risk in our male cohort. In a recent case-control study in the US (utilizing neighborhood controls), tea consumption was negatively but not significantly associated with pancreatic cancer (Mack *et al.*, 1986). Another American case-control study showed a slightly negative, but nonsignificant association in men (MacMahon *et al.*, 1981). In contrast, a recent case-control study using data from England and Wales in the 1950s showed a positive association of tea intake and risk of pancreatic cancer in men (Kinlen & McPherson, 1984). It should be noted however that these last two case-control studies used hospital controls, some (MacMahon *et al.*, 1981) or all (Kinlen & McPherson, 1984) of whom were (non-pancreatic) cancer patients. Potential selection factors among the controls in these hospital-based studies may have accounted for the difference in their findings.

Our lack of association of black tea consumption and bladder cancer risk is consistent with the results for men in several other case-control studies. The large nationwide case-control study in the USA (Hartge *et al.*, 1983) found only slightly elevated bladder cancer risk (RR = 1.2) among men consuming $>14$ cups of tea per week ($>2$ cups per day). The statistical significance of that RR was not reported. Chances of finding an association should have been very good however, since that study included more than 2,200 male bladder cancer cases and more than 4,100 male controls. Two hospital-based case-control studies found no significant differences in tea intake between bladder cancer cases and controls (Morgan & Jain, 1974; Sullivan, 1982). The remaining studies (Miller *et al.*, 1978; Howe *et al.*, 1980) found no association between the very limited tea drinking categories of ever drank/never drank and male bladder cancer risk. In these two studies, men who had ever consumed tea had RR of 1.1 and 1.0, respectively (Miller *et al.*, 1978; Howe *et al.*, 1980). Based on these past studies, there appears to be no support for an association of tea intake and bladder cancer risk in men.

The results of animal studies (Korpassy & Mosonyi, 1950; Kirby, 1960) of tannins and liver cancer were not supported by our weak negative association of black tea consumption and male liver cancer risk. This is based on only 25 cases, but is monotonic over three consumption categories. We are unaware of any epidemiologic studies relating tea intake to liver cancer risk. In the geographic correlation study (Stocks, 1970), tea consumption was negatively (but not significantly) associated with liver cancer mortality. Clearly, larger case series are required to yield more definitive results on this question.

An important confounder in any negative association of black tea and (prostate or liver) cancer risk is age, since black tea intake is also negatively related to age (Table I). Despite age-adjustment in all Cox models of risk, we may not have completely controlled for age influences. Hence, increased black tea intake might simply be a marker for younger men who are at lower prostate (or liver) cancer risk anyhow. However, the negative association of black tea and prostate cancer was observed for both younger and older men (Table IV). So, black tea consumption would not appear to be just a mask for age-related risk of prostate cancer. This age confounding possibility does not account for the strong positive association with rectal cancer risk however.

Although black tea consumption was higher among the younger men in our cohort, the overall strong positive association with rectal cancer risk was confined to the older men (Table IV). What other confounders might need consideration, expecially among the older men? Aside from age, the only other significant risk factor for rectal cancer identified to date in this cohort is alcohol intake, as reported previously (Pollack *et al.*, 1984). However, adjustment for alcohol intake in all Cox models of rectal cancer risk failed to account for the positive association with black tea intake. Saturated fat intake (as a percentage of total calories) shows a weak positive association with rectal cancer risk in this cohort (Stemmerman *et al.*, 1984). Adjusting for this factor (in addition to age and alcohol intake) had a negligible effect on the

RRs of rectal cancer at any black tea consumption level. This held whether all subjects were included, or just the older men (see Tables III & IV).

We examined (Spearman rank-type) correlations of black tea intake category with 15 potential confounding variables measured at the initial examination. This was done for all men in the analysis, and for only the men $\geqq 58$ years of age at examination. Except for the negative correlation ($r=-0.12$) of black tea intake with age at examination, the only significant ($P<0.05$) correlations among the older men were with: height ($r=0.05$), weight ($r=0.09$), pack-years of cigarette smoking ($r=-0.06$), and physical activity level ($r=-0.09$). None of these four factors have been shown to be related to rectal cancer risk in our cohort. Thus, we are unable to account for the black tea/rectal cancer association in terms of these potential confounders.

If black tea increases rectal cancer risk, the mechamism of action is not apparent. Tea is mutagenic and does contain tannins. It could have carcinogenic effects on selected organs or it might act only in the presence of other promoting factors.

In conclusion, we view our findings on black tea and cancer incidence with caution. For the strongest associations, rectal cancer (positive) and prostate cancer (negative), few if any other epidemiologic studies are available for comparison. Increased black tea consumption was not associated with higher cancer risk at any site except the rectum. We believe this observation deserves further research by others.

We thank the Honolulu Heart Program for use of its data, and the following institutions for their cooperation: Castle Medical Center, Kaiser Medical Center, Leahi Hospital, Queen's Medical Center, St. Francis Hospital, Straub Clinic and Hospital, Tripler Army Medical Center, Wahiawa General Hospital, and the Hawaii Tumor Registry. We also thank Anne Tome for data assembly and analysis; and Louise Suzuki, Minnie Hirata for typing this manuscript.

Supported by Grant R01 CA 33644 from the National Cancer Institute, National Institutes of Health, DHHS, Bethesda, MD.

### References

ARMSTRONG, B., GARROD, A. & DOLL, R. (1976). A retrospective study of renal cancer with special reference to coffee and animal protein consumption. *Br. J. Cancer*, **33**, 127.

BICHEL, J. & BACH, A. (1968). Investigation on the toxicity of small chronic doses of tannic acid with special reference to possible carcinogenicity. *Acta. Pharmacol. Toxicol.*, **26**, 41.

BOKUCHAVA, M.A. & SKOBELEVA, N.I. (1980). The biochemistry and technology of tea manufacture. *CRC Crit. Rev. Food Sci. Nutr.*, **12**, 303.

COX, D.R. (1972). Regression models and life tables (with discussion). *J. Roy. Statist. Soc.*, Series B, **34**, 187.

FREUND, R.J. & LITTELL, R.C. (1981). *Statistical analysis system (SAS) for linear models*. p. 187. SAS Institute Inc., Cary, NC.

HARRELL, F. (1983). The PHGLM procedure. In *SAS supplemental library user's guide*. p. 267. SAS Institute Inc., Cary, NC.

HARTGE, P., HOOVER, R., WEST, D.W. & LYON, J.L. (1983). Coffee drinking and risk of bladder cancer. *J. Natl Cancer Inst.*, **70**, 1021.

HOWE, G.R., BURCH, J.D., MILLER, A.B. & 7 others. (1980). Tobacco use, occupation, coffee, various nutrients, and bladder cancer. *J. Natl Cancer Inst.*, **63**, 701.

KAISER, H.E. (1967). Cancer-promoting effects of phenols in tea. *Cancer*, **20**, 614.

KAPADIA, G.J., PAUL, B.D., CHUNG, E.B., GHOSH, B. & PRADHAN, S.N. (1976). Carcinogenicity of Camellia sinensis (tea) and some tannin-containing folk medicinal herbs administered subcutaneously in rats. *J. Natl Cancer Inst.*, **57**, 207.

KINLEN, L.J. & McPHERSON, K. (1984). Pancreas cancer and coffee and tea consumption: A case-control study. *Br. J. Cancer*, **49**, 93.

KIRBY, K.S. (1960). Induction of tumours by tannin extracts. *Br. J. Cancer*, **14**, 147.

KORPASSY, B. & MOSONYI, M. (1950). The carcinogenic activity of tannic acid. Liver tumours induced in rats by prolonged subcutaneous administration of tannic acid solutions. *Br. J. Cancer*, **4**, 411.

LEE, E.T. (1980). *Statistical methods for survival data analysis*. p. 306. Lifetime Learning Publications, Belmont, CA.

MACK, T.M. (1986). Pancreas cancer and smoking, beverage consumption, and past medical history. *J. Natl Cancer Inst.*, **76**, 49.

MacMAHON, B., YEN, S., TRICHOPOULOS, D., WARREN, K. & NARDI, G. (1981). Coffee and cancer of the pancreas. *New Engl. J. Med.*, **304**, 630.

McLAUGHLIN, J.K., BLOT, W.J., MANDEL, J.S., SCHUMAN, L.M., MEHL, E.S. & FRAUMENI, Jr. J.F. (1983). Etiology of cancer of the renal pelvis. *J. Natl Cancer Inst.*, **71**, 287.

McLAUGHLIN, J.K., MANDEL, J.S., BLOT, W.J., SCHUMAN, L.M., MEHL, E.S. & FRAUMENI, Jr. J.F. (1984). A population-based case-control study of renal cell carcinoma. *J. Natl Cancer Inst.*, **72**, 275.

MILLER, A.B., HOWE, G.R., JAIN, M., CRAIB, K.J.P. & HARRISON, L. (1983). Food items and food groups as risk factors in a case-control study of diet and colorectal cancer. *Int. J. Cancer*, **32**, 155.

MILLER, C.T., NEUTEL, C.I., NAIR, R.C., MARRETT, L.D., LAST, J.M. & COLLINS, W.E. (1978). Relative importance of risk factors in bladder carcinogenesis. *J. Chronic Dis.*, **31**, 51.

MORGAN, R.W. & JAIN, M.G. (1974). Bladder cancer: Smoking, beverages and artificial sweeteners. *Can. Med. Assoc. J.*, **111**, 1067.

NAGAO, M., TAKAHASHI, Y., YAMANAKA, H. & SUGIMURA, T. (1979). Mutagens in coffee and tea. *Mutation Res.*, **68**, 101.

NEW ENCYCLOPAEDIA BRITANNICA, 15th Ed. (1979). Macropaedia Vol. **18**, p. 17. H.H. Benton: Chicago.

PHILLIPS, R.L. & SNOWDON, D.A. (1985). Dietary relationships with fatal colorectal cancer among Seventh-Day Adventists. *J. Natl Cancer Inst.*, **74**, 307.

POLLACK, E.S., NOMURA, A.M.Y., HEILBRUN, L.K., STEMMERMANN, G.N. & GREEN, S.B. (1984). Prospective study of alcohol consumption and cancer. *New Engl. J. Med.*, **310**, 617.

STEMMERMANN, G.N., NOMURA, A.M.Y. & HEILBRUN, L.K. (1984). Dietary fat and the risk of colorectal cancer. *Cancer Res.*, **44**, 4633.

STOCKS, P. (1970). Cancer mortality in relation to national consumption of cigarettes, solid fuel, tea and coffee. *Br. J. Cancer*, **24**, 215.

SULLIVAN, J.W. (1982). Epidemiologic survey of bladder cancer in greater New Orleans. *J. Urology*, **128**, 281.

TAJIMA, K. & TOMINAGA, S. (1985). Dietary habits and gastrointestinal cancers: A comparative case-control study of stomach and large intestinal cancers in Nagoya, Japan. *Jap. J. Cancer Res.*, **76**, 705.

TILLOTSON, J.L., KATO, H., NICHAMAN, M.Z. & 4 others (1973). Epidemiology of coronary heart disease and stroke in Japanese men living in Japan, Hawaii, and California: Methodology for comparison of diet. *Am. J. Clin. Nutr.*, **26**, 177.

WICKREMASINGHE, R.L. (1978). Tea. *Adv. Food Res.*, **24**, 229.

WORTH, R.M. & KAGAN, A. (1970). Ascertainment of men of Japanese ancestry in Hawaii through World War II Selective Service Registration. *J. Chron. Dis.*, **23**, 389.


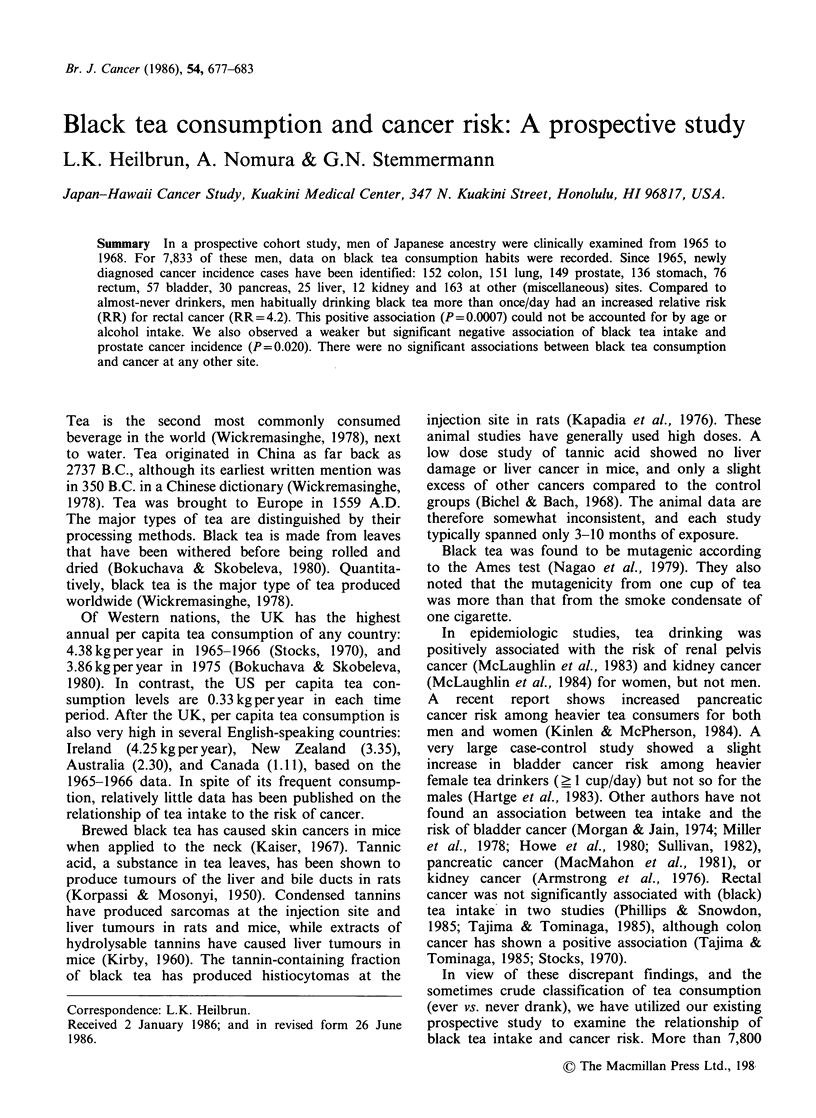

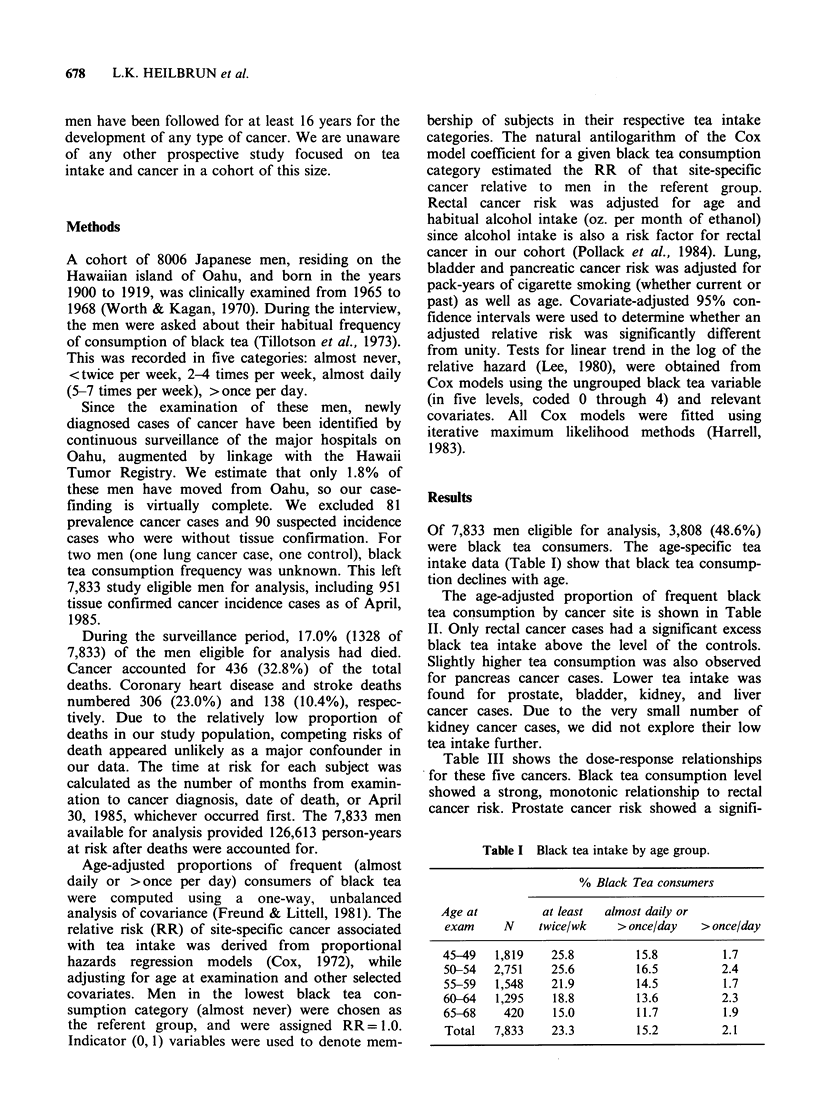

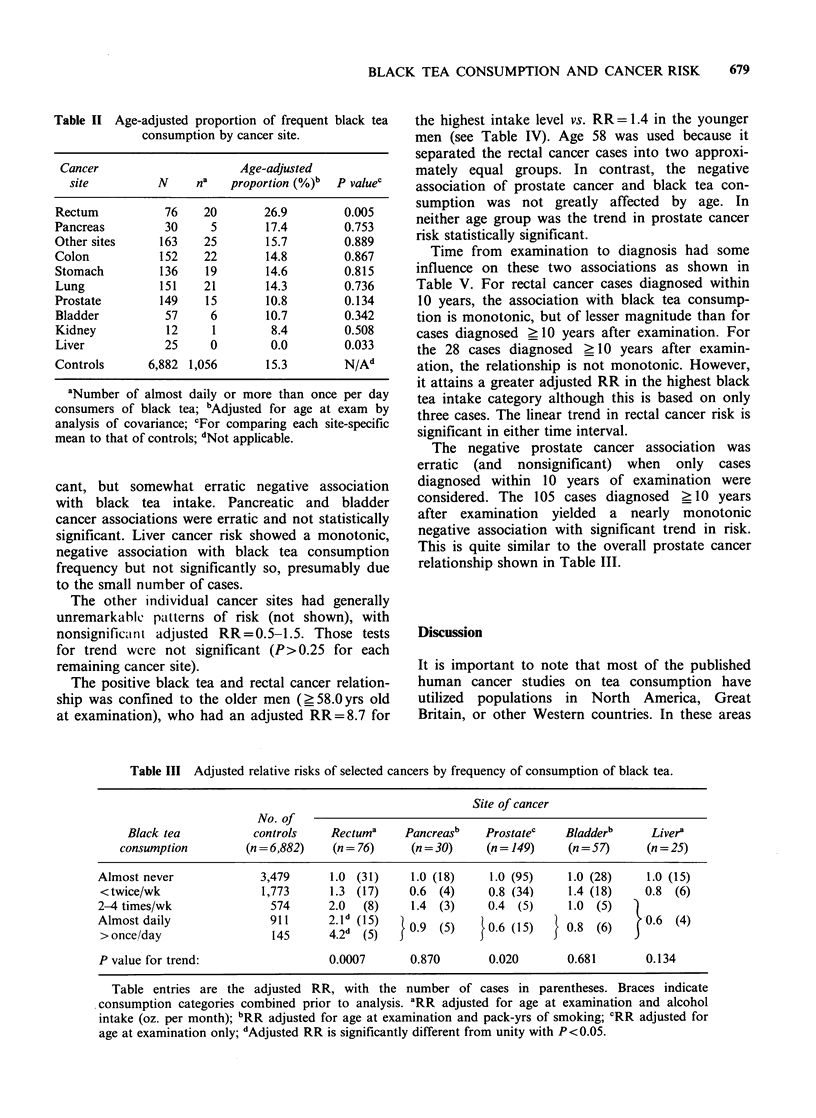

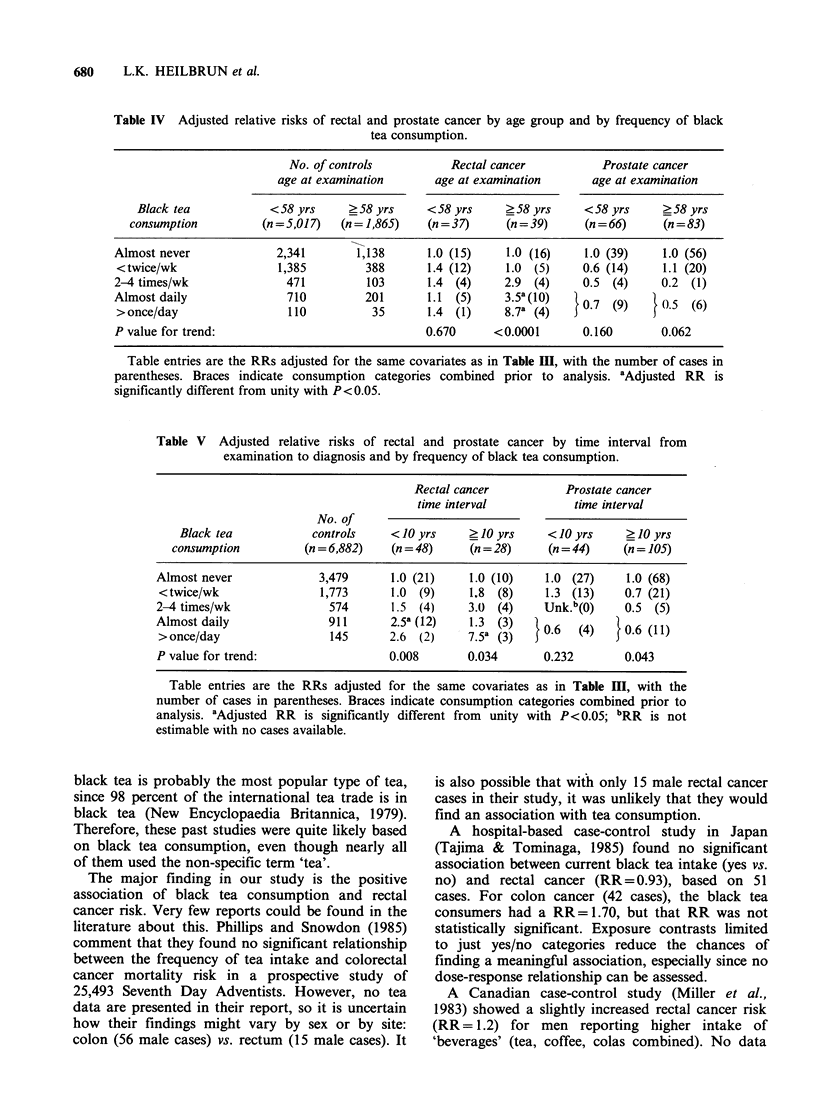

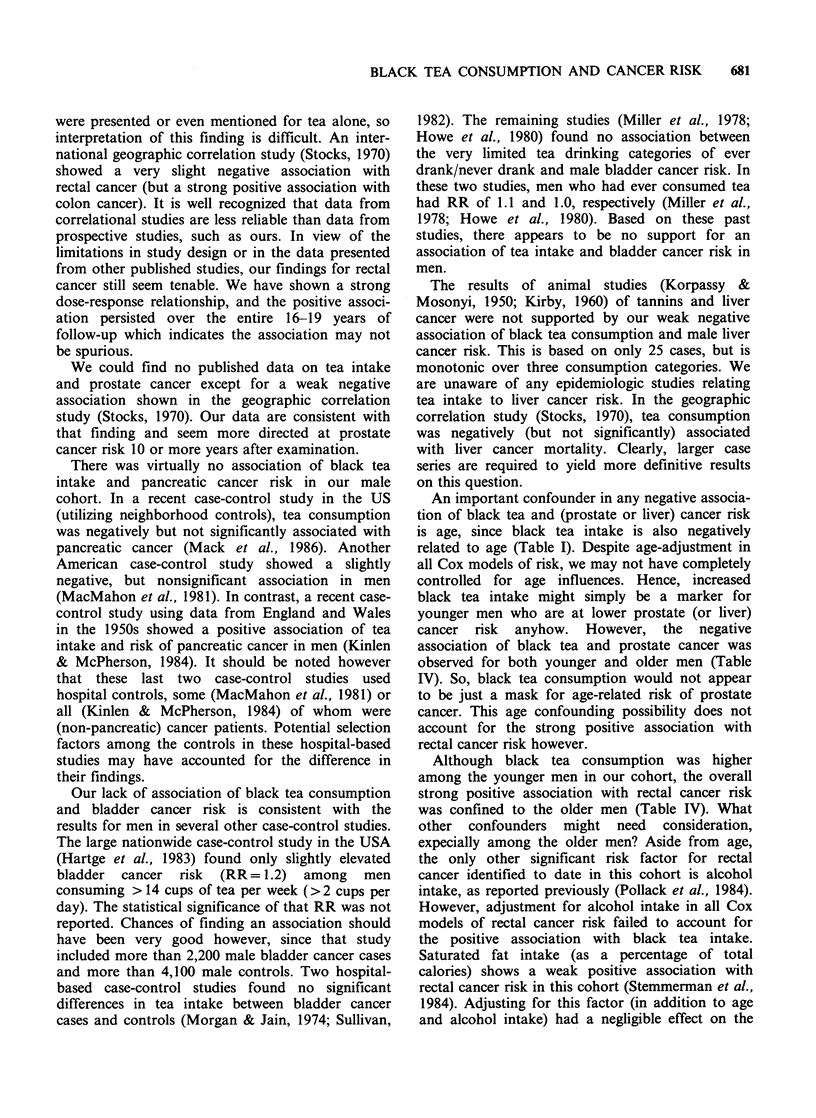

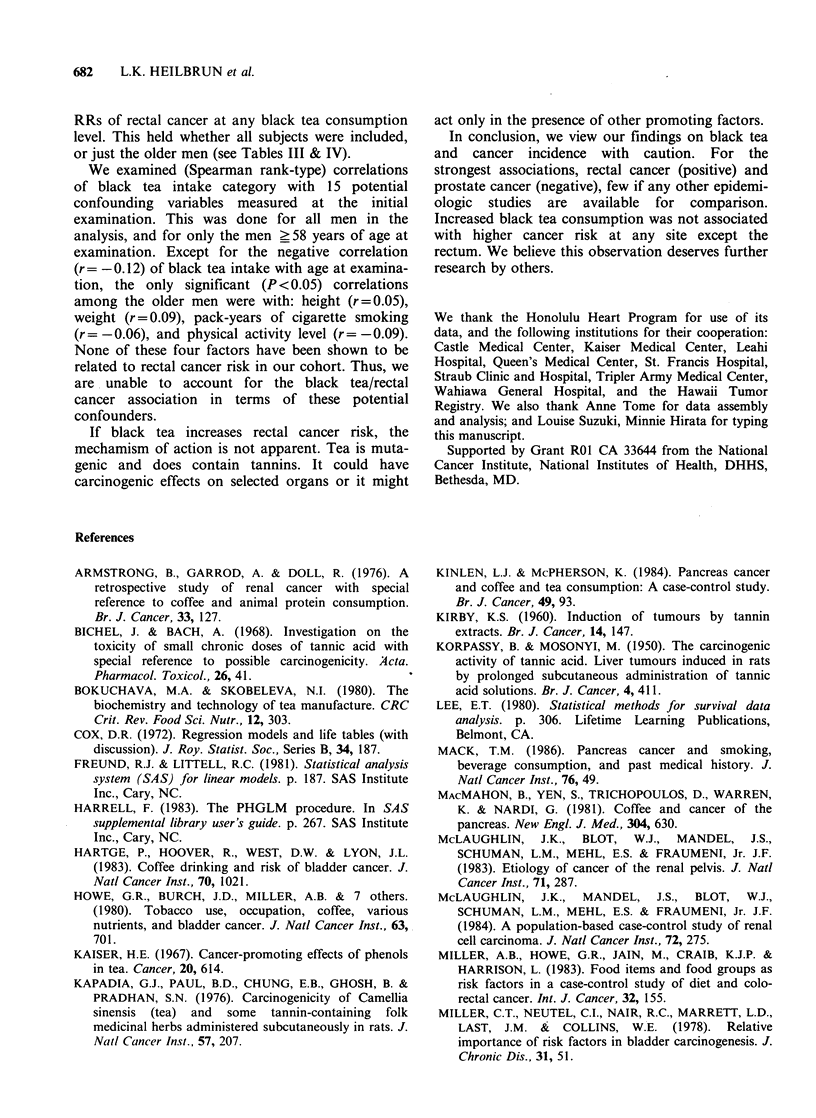

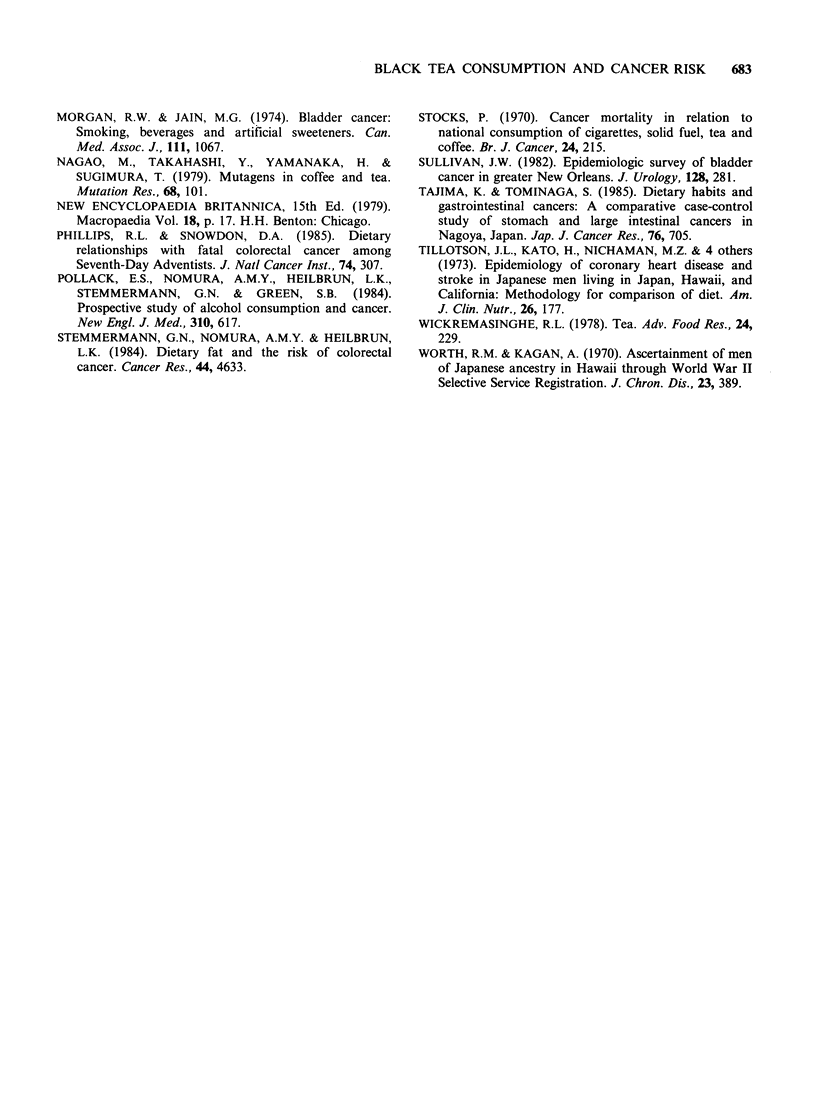


## References

[OCR_00594] Armstrong B., Garrod A., Doll R. (1976). A retrospective study of renal cancer with special reference to coffee and animal protein consumption.. Br J Cancer.

[OCR_00600] Bichel J., Bach A. (1968). Investigation on the toxicity of small chronic doses of tannic acid with special reference to possible carcinogenicity.. Acta Pharmacol Toxicol (Copenh).

[OCR_00606] Bokuchava M. A., Skobeleva N. I. (1980). The biochemistry and technology of tea manufacture.. Crit Rev Food Sci Nutr.

[OCR_00625] Hartge P., Hoover R., West D. W., Lyon J. L. (1983). Coffee drinking and risk of bladder cancer.. J Natl Cancer Inst.

[OCR_00630] Howe G. R., Burch J. D., Miller A. B., Cook G. M., Esteve J., Morrison B., Gordon P., Chambers L. W., Fodor G., Winsor G. M. (1980). Tobacco use, occupation, coffee, various nutrients, and bladder cancer.. J Natl Cancer Inst.

[OCR_00652] KIRBY K. S. (1960). Induction of tumours by tannin extracts.. Br J Cancer.

[OCR_00656] KORPASSY B., MOSONYI M. (1950). The carcinogenic activity of tannic acid; liver tumours induced in rats by prolonged subcutaneous administration of tannic acid solutions.. Br J Cancer.

[OCR_00636] Kaiser H. E. (1967). Cancer-promoting effects of phenols in tea.. Cancer.

[OCR_00640] Kapadia G. J., Paul B. D., Chung E. B., Ghosh B., Pradhan S. N. (1976). Carcinogenicity of Camellia sinensis (tea) and some tannin-containing folk medicinal herbs administered subcutaneously in rats.. J Natl Cancer Inst.

[OCR_00647] Kinlen L. J., McPherson K. (1984). Pancreas cancer and coffee and tea consumption: a case-control study.. Br J Cancer.

[OCR_00672] MacMahon B., Yen S., Trichopoulos D., Warren K., Nardi G. (1981). Coffee and cancer of the pancreas.. N Engl J Med.

[OCR_00667] Mack T. M., Yu M. C., Hanisch R., Henderson B. E. (1986). Pancreas cancer and smoking, beverage consumption, and past medical history.. J Natl Cancer Inst.

[OCR_00677] McLaughlin J. K., Blot W. J., Mandel J. S., Schuman L. M., Mehl E. S., Fraumeni J. F. (1983). Etiology of cancer of the renal pelvis.. J Natl Cancer Inst.

[OCR_00683] McLaughlin J. K., Mandel J. S., Blot W. J., Schuman L. M., Mehl E. S., Fraumeni J. F. (1984). A population--based case--control study of renal cell carcinoma.. J Natl Cancer Inst.

[OCR_00689] Miller A. B., Howe G. R., Jain M., Craib K. J., Harrison L. (1983). Food items and food groups as risk factors in a case-control study of diet and colo-rectal cancer.. Int J Cancer.

[OCR_00695] Miller C. T., Neutel C. I., Nair R. C., Marrett L. D., Last J. M., Collins W. E. (1978). Relative importance of risk factors in bladder carcinogenesis.. J Chronic Dis.

[OCR_00703] Morgan R. W., Jain M. G. (1974). Bladder cancer: smoking, beverages and artificial sweeteners.. Can Med Assoc J.

[OCR_00708] Nagao M., Takahashi Y., Yamanaka H., Sugimura T. (1979). Mutagens in coffee and tea.. Mutat Res.

[OCR_00717] Phillips R. L., Snowdon D. A. (1985). Dietary relationships with fatal colorectal cancer among Seventh-Day Adventists.. J Natl Cancer Inst.

[OCR_00722] Pollack E. S., Nomura A. M., Heilbrun L. K., Stemmermann G. N., Green S. B. (1984). Prospective study of alcohol consumption and cancer.. N Engl J Med.

[OCR_00728] Stemmermann G. N., Nomura A. M., Heilbrun L. K. (1984). Dietary fat and the risk of colorectal cancer.. Cancer Res.

[OCR_00733] Stocks P. (1970). Cancer mortality in relation to national consumption of cigarettes, solid fuel, tea and coffee.. Br J Cancer.

[OCR_00738] Sullivan J. W. (1982). Epidemiologic survey of bladder cancer in greater New Orleans.. J Urol.

[OCR_00742] Tajima K., Tominaga S. (1985). Dietary habits and gastro-intestinal cancers: a comparative case-control study of stomach and large intestinal cancers in Nagoya, Japan.. Jpn J Cancer Res.

[OCR_00748] Tillotson J. L., Kato H., Nichaman M. Z., Miller D. C., Gay M. L., Johnson K. G., Rhoads G. G. (1973). Epidemiology of coronary heart disease and stroke in Japanese men living in Japan, Hawaii, and California: methodology for comparison of diet.. Am J Clin Nutr.

[OCR_00759] Worth R. M., Kagan A. (1970). Ascertainment of men of Japanese ancestry in Hawaii through World War II Selective Service registration.. J Chronic Dis.

